# A re-evaluation of diastatic *Saccharomyces cerevisiae* strains and their role in brewing

**DOI:** 10.1007/s00253-020-10531-0

**Published:** 2020-03-13

**Authors:** Kristoffer Krogerus, Brian Gibson

**Affiliations:** VTT Technical Research Centre of Finland Ltd, Tietotie 2, P.O. Box 1000, FI-02044 VTT Espoo, Finland

**Keywords:** Yeast, Beer, Dextrin, Starch, Diastatic, Genome

## Abstract

**Abstract:**

Diastatic strains of *Saccharomyces cerevisiae* possess the unique ability to hydrolyze and ferment long-chain oligosaccharides like dextrin and starch. They have long been regarded as important spoilage microbes in beer, but recent studies have inspired a re-evaluation of the significance of the group. Rather than being merely wild-yeast contaminants, they are highly specialized, domesticated yeasts belonging to a major brewing yeast lineage. In fact, many diastatic strains have unknowingly been used as production strains for decades. These yeasts are used in the production of traditional beer styles, like saison, but also show potential for creation of new beers with novel chemical and physical properties. Herein, we review results of the most recent studies and provide a detailed account of the structure, regulation, and functional role of the glucoamylase-encoding *STA1* gene in relation to brewing and other fermentation industries. The state of the art in detecting diastatic yeast in the brewery is also summarized. In summary, these latest results highlight that having diastatic *S. cerevisiae* in your brewery is not necessarily a bad thing.

**Key Points:**

*•Diastatic S. cerevisiae strains are important spoilage microbes in brewery fermentations.*

*•These strains belong to the ‘Beer 2’ or ‘Mosaic beer’ brewing yeast lineage.*

*•Diastatic strains have unknowingly been used as production strains in breweries.*

*•The STA1-encoded glucoamylase enables efficient maltotriose use.*

**Electronic supplementary material:**

The online version of this article (10.1007/s00253-020-10531-0) contains supplementary material, which is available to authorized users.

## Introduction

Diastatic strains of *Saccharomyces cerevisiae* are an important group of spoilage microbes in industrial beer fermentations. These yeasts, in contrast to other *S. cerevisiae* strains, are capable of producing an extracellular glucoamylase enzyme that enables fermentation on oligosaccharides and starch if present. If these diastatic strains contaminate beer, they are able to release glucose from the non-reducing end of any residual oligosaccharides, which in turn leads to extended fermentation. This can have numerous negative effects on the beer, including increased carbon dioxide and ethanol levels, drier mouthfeel, and production of off-flavor, particularly the clove-like 4-vinyl guaiacol. In extreme cases, this can lead to gushing and exploding packages, endangering the consumer. Instances of contamination by diastatic yeast in commercial breweries have been reported in the USA and across Europe, and they appear to have increased in recent years (Begrow 2017; Meier-Dörnberg et al. 2017). Contaminations in the bottling area were the most common in a recent survey (Meier-Dörnberg et al. 2017). Smaller breweries in particular tend to be more susceptible to contamination, as beers are seldom pasteurized, quality control is less stringent, and experimentation with different yeast strains is more common.

Extracellular glucoamylolytic activity in yeast was observed already in the 1940s (Bishop and Whitley 1943), while the first isolation of a diastatic *Saccharomyces* strain was reported in 1952 (Andrews and Gilliland 1952). The authors proposed the taxonomic name *Saccharomyces diastaticus* and justified its classification as a separate species based on its unique phenotypic property (Gilliland 1966). Later, however, *S. diastaticus* was designated *S. cerevisiae* (as var. *diastaticus*) based on its ability to interbreed and produce fertile hybrids with *S. cerevisiae* (Tamaki 1978). This classification has now been confirmed by whole-genome sequencing (Liti et al. 2009; Peter et al. 2018). Despite this, the name *S. diastaticus* is still commonly used, particularly in the brewing industry. Interestingly, using data from recent large-scale whole-genome sequencing studies (Gallone et al. 2016; Peter et al. 2018), it has been revealed that diastatic *S. cerevisiae* contaminants belong to a lineage of brewing yeast strains (the ‘Beer 2’ or ‘Mosaic beer’ group) and many diastatic *S. cerevisiae* strains are intentionally used as production strains (Krogerus et al. 2019).

The extracellular glucoamylase associated with diastatic *S. cerevisiae* is encoded by the *STA* genes (Tamaki 1978; Yamashita et al. 1985a, b). A number of highly homologous genes, *STA1-STA3*, have been described (Tamaki 1978; Yamashita et al. 1985b; Lambrechts et al. 1991). The DNA sequences of these genes are nearly identical, but they were given different names as they were located on different chromosomes and linkage groups (Tamaki 1978). Using pulsed-field gel electrophoresis, *STA* genes have been identified on chromosomes II, IV, and XIV (Pretorius and Marmu 1988; Bignell and Evans 1990). In addition to these, long-read sequencing data also suggests that *STA1* can be found on chromosomes IX and X, in the *S. cerevisiae* A-81062 and WLP570 ‘Beer 2’ strains, respectively (Krogerus et al. 2019). Genome assemblies from the same long-read sequencing data reveal that *STA1* is located in the subtelomeric regions. These regions are known to be dynamic and unstable and are susceptible to structural rearrangements and reshuffling (Brown et al. 2010; Yue et al. 2017). In addition to *STA*, the names *DEX1* and *DEX2* have been used to describe genes encoding extracellular glucoamylases in diastatic *S. cerevisiae*, but these were shown to be allelic to the *STA* genes (Erratt and Stewart 1981; Erratt and Nasim 1986). The name *STA1* will be used throughout the rest of this mini-review to address the gene(s) encoding the extracellular glucoamylase in diastatic *S. cerevisiae*.

## Structure and formation of the *STA1* gene

**Fig. 1:**
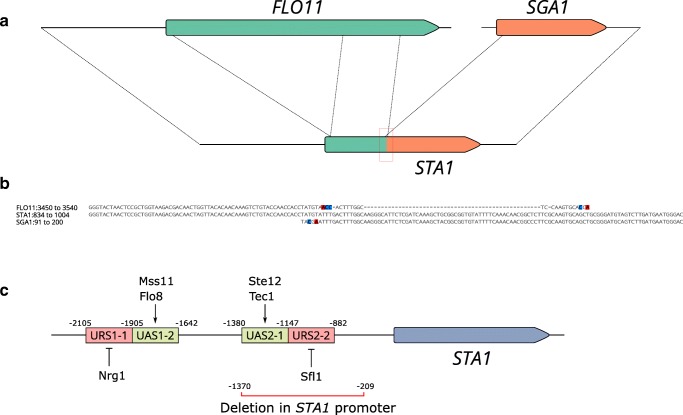
**a** The structure of *STA1* and homology to ancestral genes *FLO11* and *SGA1*. The *FLO11/SGA1* junction is highlighted with a red box, and the sequence is shown in panel **B**. The illustration is drawn to scale. **b** Multiple sequence alignment of the nucleotide sequence around the *FLO11/SGA1* junction in *STA1* (X02649.1 in the NCBI-Nucleotide database) with *FLO11* and *SGA1*. Colors indicate mutations in comparison with the *STA1* sequence. **c** The structure of the *STA1* promoter. Upstream activating sequences (UAS1-2 and UAS2-1) are shown with green boxes, and the respective activator proteins are shown above the boxes. Upstream repressing sequences (URS1-1 and URS2-2) are shown with red boxes, and the respective repressor proteins are shown below the boxes. The location of the 1162-bp deletion in the *STA1* promoter, which is common in many *STA1+* strains, is highlighted with the red line. The illustration is not drawn to scale

After the gene responsible for the diastatic phenotype was discovered, it was revealed that *STA1* appeared to be chimeric (Fig. 1a), resulting from the fusion of *FLO11* and *SGA1* (Yamashita et al. 1987; Lo and Dranginis 1996). These genes are located on opposite ends of chromosome IX in *S. cerevisiae*. The 3′ end of *STA1* is homologous to *SGA1*, a gene encoding an intracellular glucoamylase that is used during sporulation. The catalytic domain of *STA1* is located in this *SGA1-*derived peptide (Adam et al. 2004). The 5′ end of *STA1*, along with the upstream region, are homologous to *FLO11*, a gene encoding a membrane-bound flocculin promoting flocculation. This *FLO11-*derived peptide allows for extracellular secretion of the *STA1-*encoded glucoamylase (Adam et al. 2004). As the upstream regions of *STA1* and *FLO11* are nearly identical as well, these two genes are largely co-regulated (Gagiano et al. 1999). The sequence around the *FLO11*/*SGA1* junction in *STA1* contains three short homologous blocks (Yamashita et al. 1987). In particular, two 8-bp stretches at the junction are shared between *FLO11* and *SGA1* (Fig. 1b). Gene fusion appears to be a result of a non-reciprocal translocation, as chromosome IX in long-read genome assemblies of *STA1+* strains appears collinear with chromosome IX of *S. cerevisiae* S288C (Krogerus et al. 2019). Such a translocation could arise from repair of a double-stranded break by recombination mediated by the homologous blocks in *FLO11* and *SGA1*, as has been proposed for gene fusion events that have been observed in laboratory evolution experiments (Dunn et al. 2013; Brouwers et al. 2019).

The catalytic domain of Sta1p is located in the *SGA1*-derived peptide. The *SGA1-*encoded glucoamylase is involved in releasing glucose from glycogen during sporulation (François and Parrou 2001). It has also been shown to hydrolyze starch (Pugh et al. 1989). The three-dimensional structure of the Sta1p glucoamylase has not been determined, but a structural model, based on the known structure of a similar glucoamylase from *Saccharomycopsis fibuligera*, suggests that the catalytic domain has a (α/α)_6_ barrel structure, with the active site being comprised of two catalytic glutamic acid residues (E521 and E770) (Adam et al. 2004). In contrast to many other fungal glucoamylases, the Sta1p glucoamylase lacks a starch-binding domain and is thus only active on soluble starch (Sauer et al. 2000; Adam et al. 2004; Latorre-García et al. 2005).

The extracellular secretion of the *STA1-*coded glucoamylase is enabled by the *FLO11*-derived sequence. The first 32 amino acids of the amino-terminal end of Sta1p form a hydrophobic leader peptide (Yamashita et al. 1985b; Yamashita et al. 1987), which when removed hinders secretion of Sta1p (Yamashita 1989) and when fused to other enzymes allows their secretion (Yamashita et al. 1986; Vanoni et al. 1989). In addition to the signal peptide, the Sta1p sequence contains a serine- and threonine-rich region which provides multiple *O*-glycosylation sites (Yamashita 1989; Adam et al. 2004). Indeed, the *STA1*-derived glucoamylase is highly glycosylated, as protein masses of 250–300 kDa have been reported (Modena et al. 1986; Kleinman et al. 1988; Adam et al. 2004). The estimated size of the Sta1p amino acid sequence is approximately 83 kDa (Adam et al. 2004). The serine- and threonine-rich region has also been proposed to facilitate secretion of the glucoamylase, as partial and full deletion of this region reduces glucoamylolytic activity and the amount of glucoamylase detected in the culture media (Yamashita 1989; Adam et al. 2004). However, the signal peptide appears to have the larger role in enabling secretion (Marín-Navarro et al. 2011). The hyperglycosylation of Sta1p could potentially influence enzyme activity as well, e.g., by stabilizing the enzyme structure (Solovicova et al. 1999), as lower culture media glucoamylase activities were observed from a *mnn9* mutant with reduced glycosylation (Adam et al. 2004). However, it remained unclear whether the decrease in activity was a result of decreased secretion to the media or decreased specific enzyme activity.

### Regulation of *STA1* transcription

The promoter regions of *STA1* and *FLO11* are very similar. The expression of these two genes is therefore induced and repressed in similar environmental conditions. The promoters of *STA1* and *FLO11* are complex (Fig. 1c), covering over 2.5-kbp and containing multiple regulatory regions; indeed, they are one of the largest promoters in *S. cerevisiae* (Rupp et al. 1999; Kim et al. 2004a, b; Barrales et al. 2008). The *STA1* promoter contains at least two segments controlling expression, UAS1 and UAS2 (Kim et al. 2004a, b). Both can be further divided into an upstream activating sequence (UAS) and upstream repressing sequence (URS); UAS1-2 and URS1-1, respectively, for UAS1, and UAS2-1 and URS2-2, respectively, for UAS2. Each of these four segments contain transcription factor binding sites. The repressors Nrg1 and Sfl1 bind to URS1-1 and URS2-2, respectively, while the activators Mss11 and Flo8 bind to UAS1-2, and activators Ste12 and Tec1 bind to UAS2-1 (Kim et al. 2004a, b) (Fig. 1c). It has been proposed that these activators bind sequentially to the promoter to enable transcription, and bound repressors prevent their binding to the UASs.

Control of *STA1* (and *FLO11*) expression is therefore complex and appears to rely on the balance of numerous transcription factors. These transcription factors are also involved in other cellular processes. Flo8, for example, is regulated by the cAMP-PKA pathway (Pan and Heitman 1999), while Ste12 and Tec1 are involved in the MAP kinase pathway (Madhani 1997). As a result of this complexity, *FLO11* expression has been used as a model for studying how signaling pathways affect gene expression (Vinod et al. 2008; Octavio et al. 2009; Brückner and Mösch 2012). Early studies on the regulation of *STA1*, also identified an apparent repressor that was designated *STA10* (Polaina and Wiggs 1983; Pardo et al. 1986). However, later studies revealed that *STA10* was just a non-functional allele of the activator-encoding *FLO8* (Kim et al. 2003). Early studies on *STA1* regulation also revealed an apparent effect of ploidy, with higher levels of *STA1* expression in haploid strains compared with diploid strains (Pretorius et al. 1986; Dranginis 1989). It was hypothesized that the a1/α2 heterodimer that forms in MATa/MATα cells, could repress *STA1*, but no evidence of direct repression has been observed (Lambrechts et al. 1994). Rather, it is likely that a1/α2 heterodimer can indirectly affect *STA1* expression by influencing the transcription factors. Ste12, one of the activators, for example, is upregulated in the presence of α-pheromone (Brückner and Mösch 2012; Cullen and Sprague 2012). Interestingly, a number of the *STA1*+ ‘Beer 2’ strains (see Supplementary Fig. S1) appear to be haploid based on homozygosity and *HO* deletion (Peter et al. 2018). These haploid strains likely show an increased diastatic activity.

In regard to environmental factors affecting *STA1* expression, the presence of glucose has been shown to repress transcription (Pretorius et al. 1986; Dranginis 1989; Kim et al. 2004b). This appears to be partly mediated by increased levels of the Nrg1 and Sfl1 repressors resulting from growth on glucose (Kim et al. 2004b). When a *STA1*+ strain was grown in rich media containing various carbon sources, the highest *STA1* mRNA levels were measured in complex media containing glycerol and ethanol, followed by those containing starch or a mixture of maltooligosaccharides (Pretorius et al. 1986). Starch therefore does not induce expression, as was also confirmed in a later study (Dranginis 1989). *STA1* expression was considerably lower in media containing glucose or sucrose, but results show that glucose does not completely inhibit *STA1* expression (Pretorius et al. 1986; Dranginis 1989). In brewery fermentation trials, glucoamylase activity was detected in the wort after 20 h, with an increase in activity as fermentation progressed (Searle and Tubb 1981). During wort fermentations with *STA1+* strains, in contrast to *STA1*-negative brewing strains, low concentrations of glucose are present throughout most of the active fermentation as a result of the amylolytic activity (Searle and Tubb 1981; Krogerus et al. 2019). It is likely that this continuous supply of glucose to diastatic yeast can considerably influence the yeast transcriptome in comparison with *STA1*-negative brewing strains, as many fermentation-related genes, e.g., maltose permeases, are repressed by glucose (Day et al. 2002).

A recent study (Krogerus et al. 2019) revealed that many of the diastatic *S. cerevisiae* strains that were screened had a 1162-bp deletion in the promoter of *STA1* (Fig. 1c)*.* This deleted region contains the UAS2-1 activating sequence with binding sites for the Ste12 and Tec1 transcription factors (Kim et al. 2004a). The presence of this deletion significantly decreased the expression of *STA1* (over 100-fold) and the ability to grow in beer and consume dextrin (Krogerus et al. 2019; Burns et al. 2020). Therefore, not all *STA1+* strains have the same spoilage potential, and detection of the gene itself is not a reliable marker for beer spoilage potential. Detection methods for diastatic *S. cerevisiae* are discussed in more detail below.

### Functional role of *STA1*

Thanks to the glucoamylolytic activity of diastatic yeast, they have a competitive advantage compared with traditional brewing strains in dextrin- or starch-rich environments where concentrations of fermentable carbon sources are low. Beer is a prime example of such an environment, and most *STA1+* strains, in contrast to ale or lager strains, are able to grow and produce carbon dioxide in fermented beer (Meier-Dörnberg et al. 2018; Krogerus et al. 2019). In addition to longer chain carbohydrates, Sta1p efficiently hydrolyzes maltotriose and other maltooligomers (Modena et al. 1986; Kleinman et al. 1988). Early wort fermentation trials with diastatic *S. cerevisiae* already hinted towards a role for Sta1p in maltotriose use, as maltotriose was depleted from the wort more quickly with diastatic strains compared with an ale brewing strain (Searle and Tubb 1981).

**Fig. 2:**
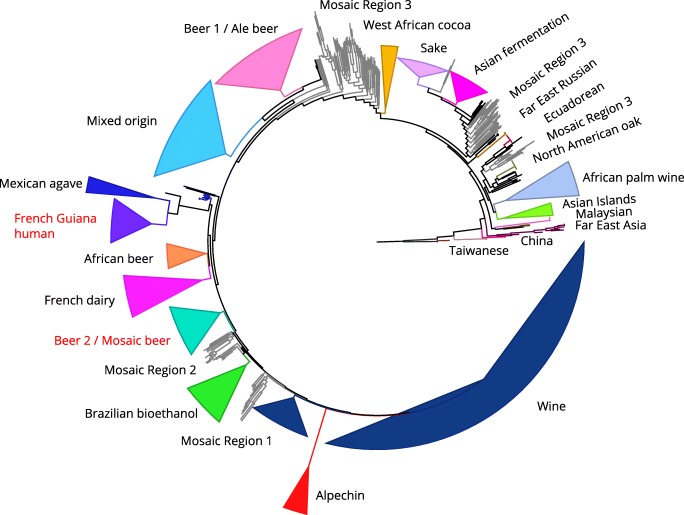
The prevalence of the *STA1* gene in 1171 publically available *Saccharomyces cerevisiae* genome assemblies (adapted from Krogerus et al. 2019). Clades have been collapsed to improve clarity and the names of clades containing *STA1*+ strains are colored red (‘French Guiana human’, and ‘Beer 2’/‘Mosaic beer’). The majority of the strains in both these clades contain *STA1*. The ‘Beer 2’ clade contains both strains isolated from contaminated beer and production strains used in commercial breweries

As discussed in the Introduction, the *STA1* gene is prevalent in the ‘Beer 2’ or ‘Mosaic beer’ lineage of *S. cerevisiae* (Fig. 2) (Gallone et al. 2016; Peter et al. 2018; Krogerus et al. 2019)*.* This lineage consists of strains isolated from and used in breweries. The other main lineage of brewing yeasts is the ‘Beer 1’ or ‘Ale beer’ lineage (Gallone et al. 2016; Gonçalves et al. 2016; Peter et al. 2018). The majority of the sequenced strains belonging to the ‘Beer 2’ lineage carry the *STA1* gene (Krogerus et al. 2019). These strains, like most brewing strains, have been shown to utilize maltotriose efficiently (Gallone et al. 2016). However, the mechanism for maltotriose use differs between the ‘Beer 2’ and the ‘Beer 1’ strains. The ‘Beer 1’ strains rely on transmembrane transport of maltotriose with the Agt1p permease, while ‘Beer 2’ strains carry frameshift mutations in the *AGT1*/*MAL11* gene (Vidgren et al. 2005; Alves et al. 2008; Duval et al. 2010; Gallone et al. 2016). ‘Beer 2’ strains rather appear to rely on extracellular hydrolysis by the Sta1p glucoamylase for maltotriose use. When *STA1* was deleted from three diastatic strains, maltotriose consumption during wort fermentations decreased significantly (Krogerus et al. 2019). Furthermore, strains carrying the 1162-bp deletion in the *STA1* promoter exhibit significantly slower maltotriose use during wort fermentations (Gallone et al. 2016; Krogerus et al. 2019). Despite extracellular hydrolysis appearing to be the dominant route of maltotriose use in diastatic strains, all three diastatic strains that were tested by Krogerus et al. (2019) still showed low transmembrane transport rates of maltotriose. This confirms that a maltotriose-transporting permease other than Agt1p is present in these strains.

Maltotriose use in brewing yeast is often considered a signature of domestication, as this sugar is fairly unique to brewer’s wort in comparison with other beverage fermentation media, and the trait is rarely found in wild yeast (Gallone et al. 2016; Gonçalves et al. 2016; Steensels et al. 2019). While this trait has previously only been attributed to maltotriose-transporting permeases, the formation and retention of the *STA1* gene appears to be an alternative evolutionary mechanism for enabling efficient use of sugars present in brewer’s wort. *STA1+* strains have likely been selected for as they are expected to have a competitive advantage in the beer environment. In addition, brewers may have inadvertently selected for these strains as they produce beer with a dry finish and mouthfeel (e.g., ‘Saison’-style beer). The diastatic trait was probably later selected against, e.g., exemplified by the spread of the 1162-bp deletion in the *STA1* promoter, which leads to a considerable decrease in expression (Krogerus et al. 2019). This counter-selection may have been driven by the desired specifications of the beer (e.g., not overly dry) or the need to store beer for extended periods without excessive buildup of pressure in vessels.

Interestingly, the *STA1* gene is also prevalent in strains belonging to the ‘French Guiana human’ population (Peter et al. 2018; Krogerus et al. 2019). These strains have been isolated from various origins including fruits, animals, and mainly human feces in a remote village in French Guiana (Angebault et al. 2013). None of these strains have been linked to beer contamination. However, a traditional starch-rich fermented beverage called cachiri is produced and consumed in the village (La Barre 1938; Carrizales et al. 1986). It is likely that this starch-rich environment has been selected for diastatic strains. The evolutionary relationship between the ‘Beer 2’ and ‘French Guiana human’ strains remains unclear, but sequence similarity of *STA1* and common polymorphisms compared with *FLO11* in strains from the two populations suggest that *STA1* originates from a common gene fusion event. Population structure analysis based on single nucleotide polymorphisms, suggests that the ‘French Guiana human’ population is a clean lineage, but the ‘Beer 2’ strains show evidence of admixture, particularly from South American bioethanol strains (Peter et al. 2018). However, the admixture analysis revealed no contribution from the ‘French Guiana human’ population in the ‘Beer 2’ strains, and further study around this topic is required.

The *STA1* gene has also been found in various *Saccharomyces* interspecies hybrids. A *S. cerevisiae* × *Saccharomyces uvarum* hybrid, ‘Muri,’ that was isolated from a traditional Norwegian farmhouse brewing yeast culture was shown to possess *STA1* (Krogerus et al. 2018). Whole-genome sequencing revealed that the *S. cerevisiae* parent strain of this hybrid belonged to the ‘Beer 2’ lineage. A genetically very similar hybrid, WLP351, was also recently identified, and it is possible that it is the same strain (Langdon et al. 2019). *STA1* is also present in the *S. cerevisiae* sub-genomes of various *S. cerevisiae* × *Saccharomyces kudriavzevii* hybrids that have been isolated from beer (Gallone et al. 2019). Again, the *S. cerevisiae* parent strains of these hybrids belong to the ‘Beer 2’ lineage.

In addition to enabling the utilization of longer chain carbohydrates, Sta1p also contributes to flocculation (Lo and Dranginis 1996). Deletion of *STA1* from a diastatic *S. cerevisiae* strain decreased flocculation ability, but not to the same extent as deletion of *FLO11* from the same strain. It is likely that this flocculating activity results from interactions between Sta1p and Flo11p, as flocculins like Flo11p are known to form aggregates (Douglas et al. 2007). Like most brewers’ yeasts, many of the *STA1*+ ‘Beer 2’ strains exhibit high levels of flocculation, which is typically a desired trait in brewery fermentations (Gallone et al. 2016).

### Detection of diastatic yeast in the brewery

**Fig. 3:**
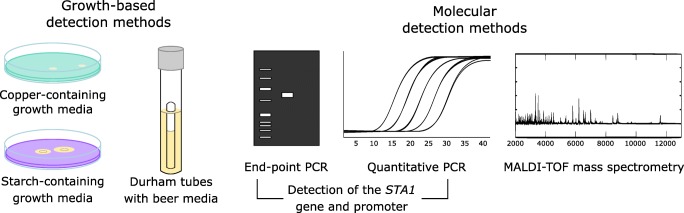
A summary of detection methods available for the detection of diastatic *S. cerevisiae* in brewery samples. The methods can broadly be divided into growth-based and molecular methods

As diastatic yeast can have considerable negative impacts on beer quality, it is vital that they can be rapidly and reliably detected within the brewery. As they are genetically and physiologically similar to brewing strains, it can be difficult to distinguish contaminants from production strains. Detection methods for beer spoilage yeast can broadly be divided into traditional growth-based methods, and modern molecular methods (Powell and Kerruish 2017). The growth-based methods rely on culturing samples on selective media that either promote the growth of the spoilage yeast or prevent the growth of the production yeast (Fig. 3). Because of their simplicity and low cost, these methods are widely used in the brewing industry. However, they typically require a long time before results are available (sometimes weeks) and may generate false positives depending on the selective media.

In the case of diastatic *S. cerevisiae*, copper-containing media are often used for detection. Such media include LCSM (Lin’s Cupric Sulphate Medium) and MYGP+copper (Malt extract, Yeast extract, Glucose, and Peptone media with copper) (Lin 1981; Taylor and Marsh 1984; van der Aa Kühle 1998). Ale and lager strains of *S. cerevisiae* are usually copper-sensitive and are unable to grow on these media, while diastatic *S. cerevisiae* strains are copper-tolerant. This tolerance has been linked to elevated copy numbers of the *CUP1* locus (Zhao et al. 2014; Peter et al. 2018). However, as selection is based on copper tolerance, rather than diastatic ability, there is a potential for false positives. Wine strains of *S. cerevisiae*, for example, also tend to exhibit high copper tolerance (see Supplementary Fig. S2; Peter et al. 2018). Media containing starch or maltodextrin as the only fermentable carbon source have also been developed (Meier-Dörnberg et al. 2018; Burns et al. 2020). Diastatic strains can grow on these media thanks to their glucoamylolytic activity. To aid with evaluation, a pH indicator such as bromophenol blue or bromocresol green can be added to the media, so that growth is accompanied by a color change. Iodine can also be used to stain residual oligosaccharides in the growth media (Burns et al. 2020). However, growth is typically very slow on starch-containing agar, often requiring several weeks, and plates should ideally be incubated anaerobically to reduce the amount of false positives (Meier-Dörnberg et al. 2018). Durham tubes containing beer as growth media have also been used successfully to detect diastatic *S. cerevisiae* (Meier-Dörnberg et al. 2018). For these, a sample of yeast is inoculated into sterilized beer inside a Durham tube, and carbon dioxide production is monitored by the buildup of gas in the inverted inner tube. As with the agar-based methods, this method can also be time-consuming.

Various molecular detection methods have also been developed for the detection of diastatic *S. cerevisiae* (Fig. 3). Compared with the traditional growth-based methods, these are rapid and can give results in hours instead of days. The most widely used rely on the detection of the *STA1* gene using polymerase chain reaction (PCR), and several commercial kits are available. DNA is extracted from the yeast sample, and the SD-5A/SD-6B primer pair, for example, can be used to detect the presence of *STA1* (Yamauchi et al. 1998). However, as was mentioned in the section on the regulation of *STA1* transcription, not all strains possessing *STA1* show spoilage potential, and this has been linked to a 1162-bp deletion in the *STA1* promoter that significantly reduces expression of the gene (Krogerus et al. 2019). Primers for detecting this deletion in the *STA1* promoter have also been developed and can be used in combination with the SD-5A/SD-6B primers to improve the reliability of detection. However, in a recent screen of 15 *STA1+* strains from the Omega Yeast Labs collection, it was revealed that contamination of bottled beer with strains possessing the deletion in the promoter can still cause some refermentation in the bottle (Burns et al. 2020). Hence, a combination of a growth-based and PCR-based method is therefore recommended to accurately differentiate spoilage and non-spoilage *STA1+* strains at the expense of time. Real-time or quantitative PCR systems have also been developed for the detection of the *STA1* gene, and the further advantage of these is that lower detection levels can be achieved and the level of contamination can also be quantified (Brandl 2006; Meier-Dörnberg et al. 2018; Schönling et al. 2019). To improve (i.e., decrease) the detection limit of these methods, a pre-enrichment in selective growth media can also be applied to the yeast sample. In addition to these PCR-based methods, MALDI-TOF mass spectrometry has also been successfully applied to separate diastatic *S. cerevisiae* from brewing strains based on proteome mass spectra (Lauterbach et al. 2017). However, this method would likely not discriminate between closely related strains with and without the *STA1* gene. While no immunoassays are, to our knowledge, available for the detection of diastatic *S. cerevisiae*, several such assays have been developed for the detection of other brewery contaminants (Nakakita et al. 2002). The direct detection of the Sta1p glucoamylase from a beer or wort sample could yield another valuable approach to detecting diastatic *S. cerevisiae* contaminants.

### Industrial applications of diastatic yeast

Despite their association with poor process hygiene and beer quality problems, diastatic strains of *S. cerevisiae* are considered to be acceptable production strains in specific cases. In such cases, the negative attributes of the group, i.e., over-attenuation and production of the phenolic flavor note 4-vinyl guaiacol, are considered positive features. In particular, diastatic strains are associated with saison-style beers, which have been growing in popularity for some years (RateBeer 2020). This farmhouse ale style, originating from Belgium, was traditionally brewed in autumn and stored until summer, when it was served to seasonal agricultural workers (saisonniers) as refreshment (Markowski 2004). Like other farmhouse ale beers not traditionally brewed for commercial purposes, the saison style is only loosely defined and there is no clear consensus regarding strength, flavor or color of the traditional saison forms. However, the modern saison is typified at least by a dry mouthfeel and spicy, phenolic character. Commercial saison starter cultures are usually diastatic strains of *S. cerevisiae* carrying the *STA1* gene.

The acceptability of diastatic *S. cerevisiae* strains is clearly dependent on the brewing context, suggesting that, under controlled conditions, their use may be helpful in developing new flavor profiles for beers. With this in mind, Meier-Dörnberg et al. (2018) carried out a comprehensive evaluation of the brewing properties of multiple diastatic strains. Many of these strains were found to possess positive characteristics from a brewing perspective. These included strong flocculation and varied ability to utilize fermentable sugars and dextrins. All strains were found to produce a phenolic note, but in sensory trials, this property was not over-powering and beers produced were considered to be acceptable. Interestingly, the yeasts differed among each other and in relation to a reference strain with respect to flavor profile. Many showed strong fruit flavors, with tropical flavors being notable in some beers. Likewise, of the 157 *S. cerevisiae* strains studied by Gallone et al. (2016), the strains in the ‘Beer 2’ clade were consistently among the top producers of several flavor-active esters, typically producing more than the ‘Beer 1’ strains (Supplementary Fig. S3). Results suggest that the genetic diversity seen within the group could be used profitably for the creation of new beers, assuming the cultures are properly handled to reduce risk of contamination. The use of diastatic strains of *S. cerevisiae* in co-culture fermentations or in controlled secondary fermentations could likewise be used to add character to beer.

The phenolic flavor compound 4-vinylguaiacol is associated with beers produced with diastatic *S. cerevisiae* strains (Russell et al. 1983)—a feature that is not negative per se, but which limits the general applicability of these strains. For example, the popular ‘M’ strain whiskey yeast is believed to be a hybrid resulting from a cross with a diastatic yeast contaminant and, as a result, produces 4-vinyl guaiacol. The phenolic flavor is however expected to be reduced during distillation, thereby negating the influence of the phenotype (Pauley and Maskell 2017). In beer, and particularly in lager beer, phenolic flavor notes can negatively influence the acceptability of the product. Indeed, many ale and lager strains lack the ability to produce phenolic off-flavors as a result of loss-of-function mutations in *PAD1* or *FDC1*, two genes which are essential for the phenolic off-flavor phenotype (Mukai et al. 2014; Gallone et al. 2016). Non-phenolic strains with diastatic potential would be more appealing to brewers interested in creating beers that have a dry mouthfeel, or which can be promoted as low-calorie beverages. Recent studies have indeed revealed that some phenolic off-flavor negative (POF-) strains exist among the commercially available *STA1*+ strains (Gallone et al. 2016; Krogerus et al. 2019). This need also inspired Tubb et al. (1981) to create diastatic/phenolic off-flavor negative (*STA1*+/POF−) strains through the use of rare mating. The procedure involved crossing a *STA1*+/POF+ strain with a *STA1*−/POF− to create a heterozygous strain containing all parental alleles, and which could subsequently by sporulated to create a *STA1*+/POF− strain through meiotic segregation. The same approach has been used recently to create POF− *Saccharomyces* hybrids from diverse parents (Krogerus et al. 2017; Nikulin et al. 2018). The strain developed by Tubb et al. (1981), had some limitations. In particular, only about 25% of beer dextrin was utilized, a result attributed to an inability of the enzyme to act on dextrin α-1,6 branch points. Also, the enzyme was active only relatively late in the fermentation. It was suggested by the authors that genetic engineering approaches could be more effective in introducing the diastatic trait without any negative effect on performance or product quality. Indeed, this approach was pursued actively in subsequent years, with several studies demonstrating the efficacy of introducing dextrin-degrading enzymes to production yeast. These included enzymes produced by genes derived from diastatic *S. cerevisiae* (Sakai et al. 1989; Latorre-García et al. 2008), but also from *Aspergillus* spp., *Debaryomyces occidentalis*, *Lipomyces* spp*.*, and *Schwanniomyces occidentalis* (Cole et al. 1988; Lancashire et al. 1989; Gopal and Hammond 1992; la Grange-Nel et al. 2004; Wang et al. 2012; Park et al. 2014). The strain created by Gopal and Hammond (1992) has arguably been the most effective, with production-scale trials showing good fermentation performance, high attenuation, and acceptable flavor profile. The strain was the first to be approved for use in preparation of food for human consumption, but has never been applied industrially due to the continued consumer skepticism regarding GMO use in foods (Hammond 1995).

For the foreseeable future, brewers that wish to utilize diastatic yeasts, but prefer to avoid the phenolic phenotype may be limited to more ‘natural’ approaches to strain development. In addition to the hybridization and genetic segregation approach taken by Tubb et al. (1981), brewers could obtain diastatic yeasts from their own process lines or from other fermentation systems (Troilo et al. 2020). The phenolic phenotype could then be eliminated from these yeasts through, for example, UV mutagenesis and screening, as has been successfully demonstrated for the wild yeast *S. eubayanus* (Diderich et al. 2018).

The ability of diastatic strains of *S. cerevisiae* to utilize dextrin is relevant not only to brewing, but to any fermentation process involving starchy raw materials. Starch is a popular substrate for bioethanol production due to its abundance and low cost, especially when derived from agricultural or industrial residues (Balat and Balat 2009). This material does, however, need to be converted to fermentable sugars to enable fermentation. An obvious advantage of diastatic *S. cerevisiae* strains is that some costs associated with the use of exogenous enzymes may be reduced. While not the only yeast group expressing glucoamylase activity, these yeasts have the additional advantage of belonging to a species associated with high and consistent productivity, resistance to ethanol toxicity and osmotolerance. Indeed, diastatic *S. cerevisiae* strains are routinely isolated from bioethanol fermentation processes as well as other processes, including brewing, that utilize starch as a starting material (Laluce and Mattoon 1984; Troilo et al. 2020). Early studies showed that these strains had good potential for bioethanol production, either in pure culture (Laluce and Mattoon 1984; Amin et al. 1985) or co-culture (Amutha and Gunasekaran 2001). A limitation of the *STA1*-encoded glucoamylase is its lack of a starch-binding domain (Latorre-García et al. 2005), meaning that it has little ability to degrade raw, non-soluble starch, even when over-expressed (Nakamura et al. 1997). This limitation has been resolved by fusing the *STA1* gene with the starch-binding domain of an *Aspergillus niger* glucoamylase gene, and expression of the gene fusion in *S. cerevisiae* (Latorre-García et al. 2005). Despite this improvement in productivity, there are other limitations, such as weak debranching activity of the enzyme (Nakamura et al. 1999). Accordingly, most attempts to engineer *S. cerevisiae* strains for glucoamylase production have involved introduction of genes from fungal species other than *S. cerevisiae* (Görgens et al. 2015). The *STA1* signal peptide that mediates the extracellular expression of the enzyme has however been exploited for the expression of other hydrolytic enzymes such as α-amylase, cellobiohydrolase, and β-glucosidase (Nam et al. 1994; Kang et al. 1996; Marín-Navarro et al. 2011; Gurgu et al. 2011; Gurgu et al. 2013).

## Conclusions

Interest towards diastatic *S. cerevisiae* has increased in recent years. This results from an increase in the number of reported contaminations, and an increase in the popularity of specialty beers. These strains have traditionally been associated with beer spoilage, particularly in the lager brewing industry, and they can cause significant quality issues in the case of contamination. However, at the same time, diastatic strains have also inadvertently been used as production strains in many breweries. Indeed, with the increasing availability of whole-genome sequence data from brewing strains, it has been revealed that diastatic *S. cerevisiae* are widespread in the ‘Beer 2’ or ‘Mosaic beer’ population, and there is little genetic distinction between contaminant and production strains. The positive traits of diastatic *S. cerevisiae* strains may have been unfairly overlooked in the past decades due to their status as spoilage yeasts. Recent studies have shown that these strains possess desirable brewing properties, particularly a high formation of flavor-active esters, and could be used for strain and product development. In addition, from an evolutionary perspective, diastatic strains offer insight on mechanisms used for adaptation to fermentation environments. The formation and retention of the chimeric *STA1* gene, which causes the diastatic phenotype, not only enables dextrin fermentation, but appears to be the main mechanism for maltotriose use in diastatic *S. cerevisiae*. This is in contrast to ale and lager brewing strains, which use the Agt1p permease to transport maltotriose into the cell. Interestingly, *STA1* is also found in multiple strains that were isolated from a remote village in French Guiana. Future studies still need to clarify the origin of *STA1* and the relatedness of *STA1+* strains. To conclude, the resurgence of interest in diastatic *S. cerevisiae* has been inspired by this group’s negative attributes, but has fortuitously revealed many positive traits that can be exploited profitably by the brewing industry.

## Electronic supplementary material

ESM 1 (PDF 368 kb)
