# Supplementary material for: A re-evaluation of diastatic Saccharomyces cerevisiae strains and their role in brewing
Source: Appl Microbiol Biotechnol. 2020 Mar 13;104(9):3745–56. doi: 10.1007/s00253-020-10531-0 (PMC7162825; doi:10.1007/s00253-020-10531-0)
Supplement: Supplementary file 1 — (PDF 368 kb) [file 253_2020_10531_MOESM1_ESM.pdf]

Supplementary Material

Journal: Applied Microbiology and Biotechnology

Title: A re-evaluation of diastatic *Saccharomyces cerevisiae* strains and their role in brewing

Kristoffer Krogerus[1#], Brian Gibson[1]

[1] VTT Technical Research Centre of Finland, Tietotie 2, P.O. Box 1000, FI-02044 VTT, Espoo, Finland

# Address correspondence to Kristoffer Krogerus, kristoffer.krogerus@gmail.com

A.

Strain	NCBI-SRA accession number	Estimated ploidy based on allele frequency distribution	Reported ploidy in Peter et al. (2018)	HO deletion as reported in Peter et al. (2018)
AAQ	ERR1309237	1	1	Yes
AAR	ERR1308657	1	1	Yes
AEA	ERR1309406	1	1	Yes
AEQ	ERR1309512	1	1	Yes
AFA	ERR1309420	1	2	No
AFB	ERR1308680	1	1	Yes
AFP	ERR1309364	1	2	No
AQG	ERR1308609	2	2	No
AQH	ERR1309146	3	3	No
Beer002	SRR5678585	2	NA	NA
Beer004	SRR5678609	2	NA	NA
Beer011	SRR5678570	2	NA	NA
Beer013	SRR5678568	2	NA	NA
Beer021	SRR5678680	2	NA	NA
Beer032	SRR5678684	2	NA	NA
Beer034	SRR5678686	1 *	NA	NA
Beer039	SRR5678681	2	NA	NA
Beer040	SRR5678682	2	NA	NA
Beer059	SRR5688171	2	NA	NA
Beer062	SRR5688177	2	NA	NA
Beer080	SRR5688213	2	NA	NA
Beer083	SRR5688219	1 *	NA	NA
Beer084	SRR5688221	1 *	NA	NA
Beer085	SRR5688223	2	NA	NA
Beer086	SRR5688225	2	NA	NA
Beer091	SRR5688235	2	NA	NA
Beer092	SRR5688237	4	NA	NA
BRM	ERR1309517	1	1	No
CFF	ERR1309102	2	2	No
Wine019	SRR5688275	1 *	NA	NA

* Strains show sporulation (Gallone et al. 2016), so are likely homozygous diploids.

B.

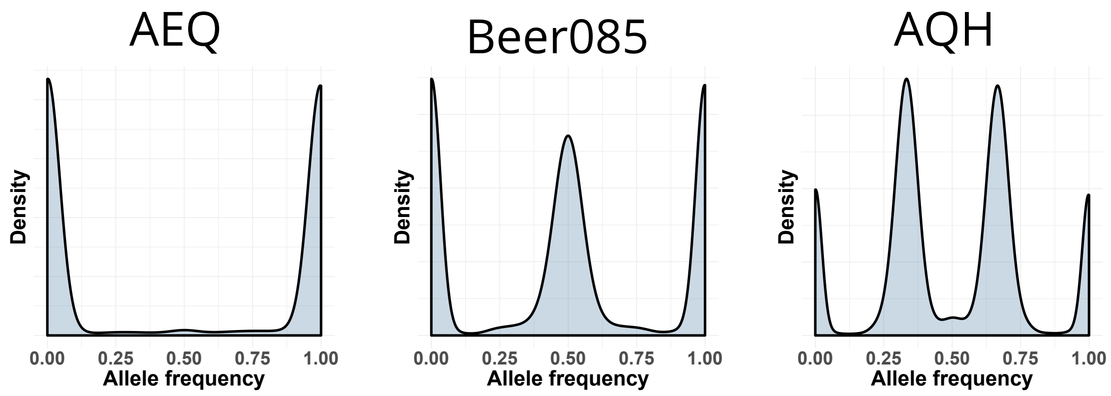

Supplementary Figure S1 – (**A**) The estimated and (when available) reported ploidy of the *STA1+* *Saccharomyces cerevisiae* strains that were sequenced in Gallone et al. (2016) and Peter et al. (2018). The ploidy was estimated based on SNP allele frequency distributions. An example of three such distributions can be seen in panel (**B**). Strain AEQ shows a haploid profile (0, 1), Beer085 a diploid profile (0, 0.5, 1), and AQH a triploid profile (0, 0.33, 0.67, 1). NA: not available.

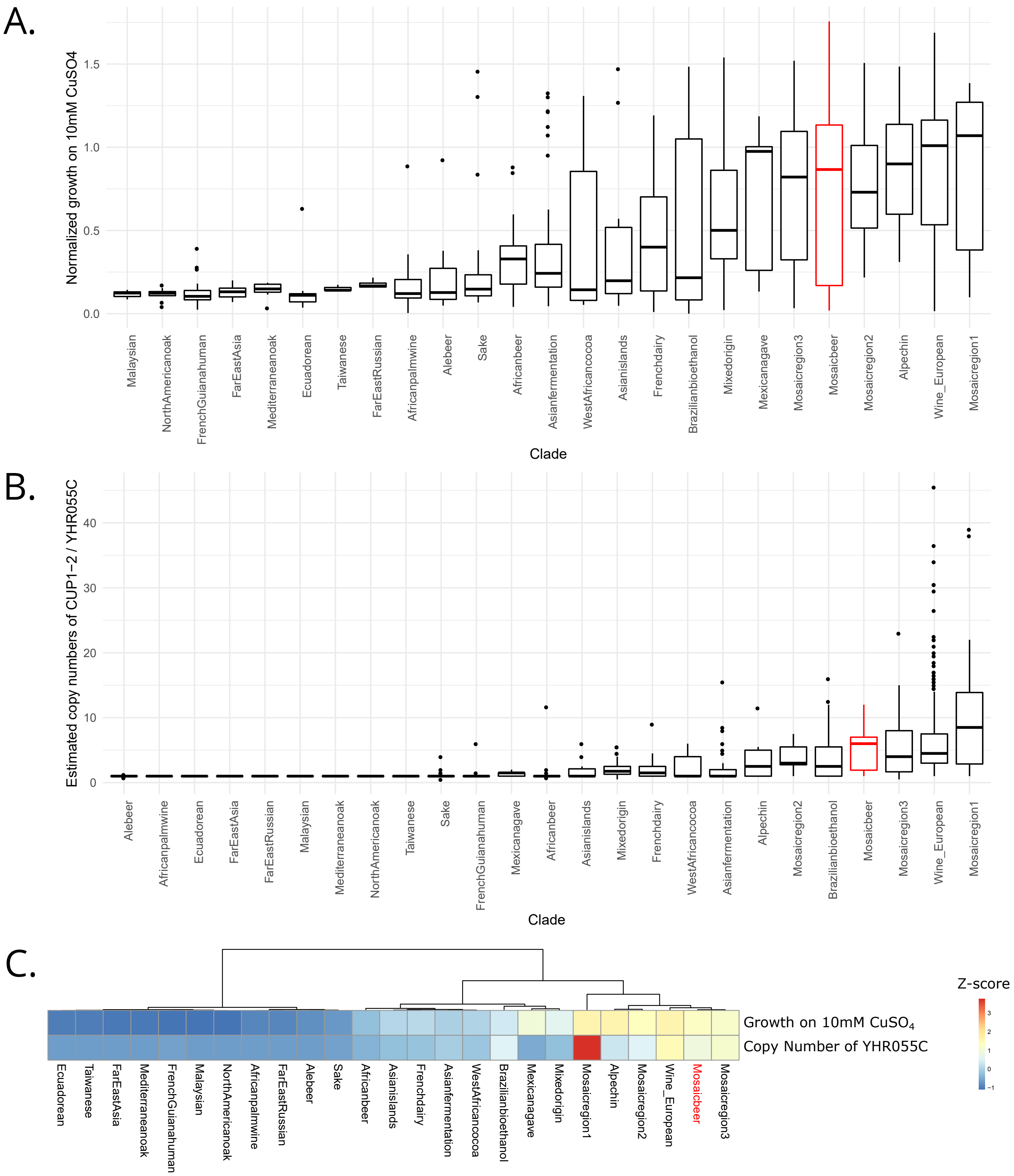

Supplementary Figure S2 – The (**A** and **C**) copper tolerance and (**B** and **C**) estimated *CUP1* copy numbers of the *S. cerevisiae* strains that were sequenced in Peter et al. (2018). Strains have been grouped by their assigned clades. The diastatic *S. cerevisiae* strains that have been isolated from contaminated beer are found in the 'Mosaic beer' clade (colored red).

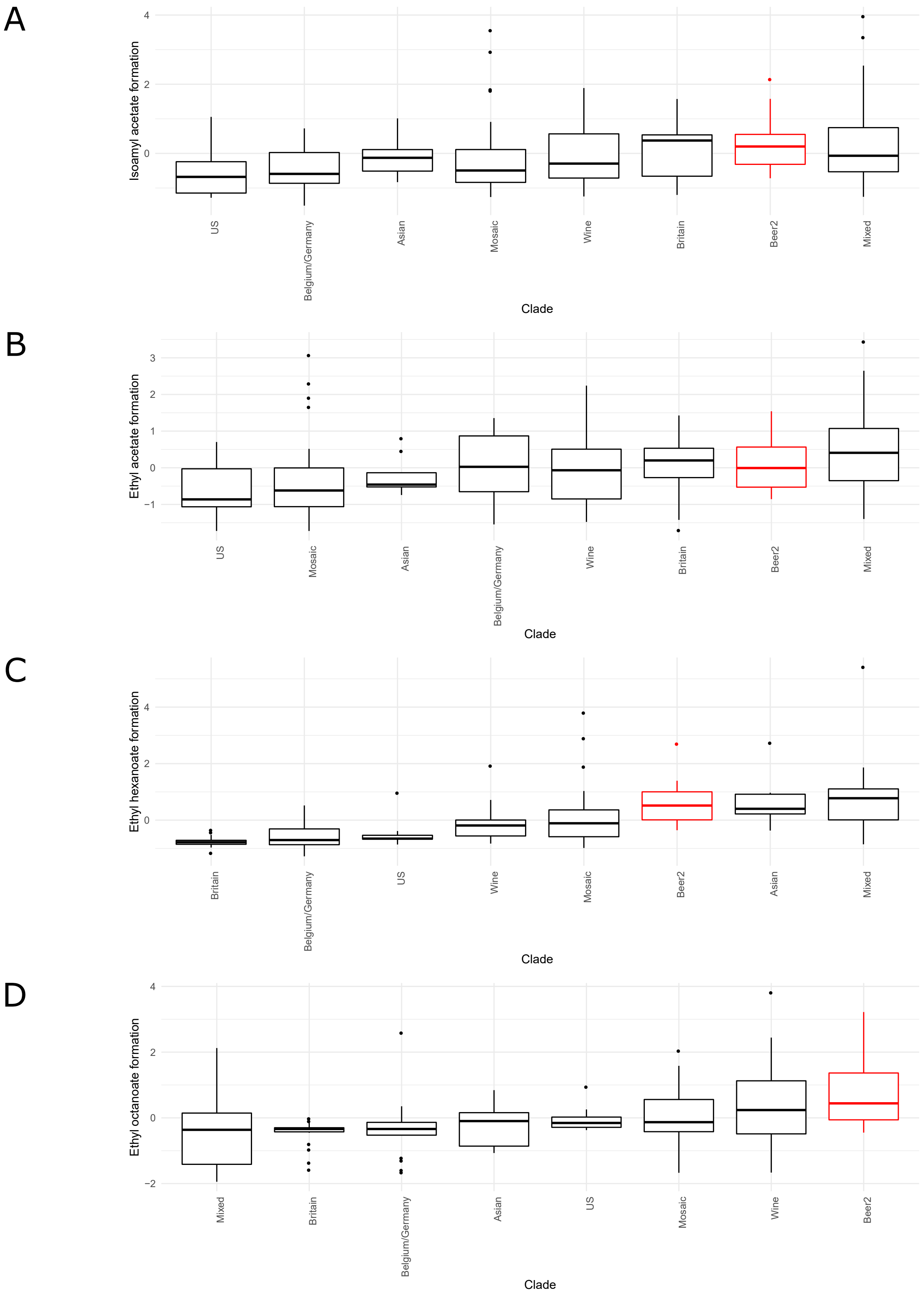

Supplementary Figure S3 – The normalized amounts (Z-scores) of flavor-active esters produced by the *S. cerevisiae* strains studied in Gallone et al. (2016). The Z-scores were obtained from Supplementary Table S5 in Gallone et al. (2016). (**A**) isoamyl acetate, (**B**) ethyl acetate, (**C**) ethyl hexanoate, and (**D**) ethyl octanoate. Strains have been grouped by their assigned clades. The diastatic *S. cerevisiae* strains are found in the 'Beer 2' clade (colored red).
